# O11.5. EFFECTIVENESS OF COORDINATED SPECIALTY CARE FOR EARLY PSYCHOSIS

**DOI:** 10.1093/schbul/sby015.265

**Published:** 2018-04-01

**Authors:** Britta Galling, Christoph Correll

**Affiliations:** 1 The Zucker Hillside Hospital; 2 The Zucker Hillside Hospital, Hofstra North Shore LIJ School of Medicine

## Abstract

**Background:**

The value of early intervention in psychosis and allocation of public resources has long been debated since outcomes in people with schizophrenia-spectrum disorders have remained suboptimal. Several research programs for early psychosis yielded promising results for team-based, multi-element coordinated specialty care (CSC).

**Methods:**

Systematic literature search of PubMed/PsycInfo/Embase/clinicaltrials.gov without language restrictions until 06/06/2017. Random effects meta-analysis of randomized trials comparing CSC versus Treatment as Usual (TAU) in in first episode psychosis or early-phase schizophrenia-spectrum disorders (schizophrenia, psychotic disorder not otherwise specified, schizoaffective disorder, schizophreniform disorder, delusional disorder), calculating standardized mean differences (SMDs) and risk ratios (RRs) for continuous and categorical outcomes as well as prespecified subgroup and meta-regression analyses.

Co-primary outcomes were all-cause treatment discontinuation and ≥1 psychiatric hospitalization during the treatment period. Key secondary outcomes were total symptom improvement, functioning, and work or school involvement.

**Results:**

Across 10 trials (n=2,176; age=27.5 ± 4.6 years; male=62.3%; trial duration=16.2 ± 7.4 (range=9–24) months), CSC outperformed TAU at the end of treatment regarding all meta-analyzable outcomes. This included all-cause discontinuation (studies=10, n=2,173, RR=0.70, 95% confidence interval (CI)=0.61–0.80, p<0.001; number-needed-to-treat (NNT)=12.4), ≥1 hospitalization (studies=10, n=2,105, RR=0.74, 95%CI=0.61–0.90, p=0.003; NNT=10.1), total symptom severity (studies=8, n=1,179, SMD=-0.32, 95%CI=-0.47, -0.17, p<0.001), positive symptoms (studies=10, n=1,532, SMD=-0.22, 95%CI=-0.32, -0.13, p<0.001), negative symptoms (studies=10, n=1,432, SMD=-0.28, 95%CI=-0.42, -0.14, p<0.001), general symptoms (studies=8, n=1,118, SMD=-0.30, 95%CI=-0.47, -0.13, p=0.001), depressive symptoms (studies=5, n=874, SMD=-0.19, 95%CI=-0.35, -0.03, p=0.017), functioning (studies=7, n=1,005, SMD=0.21, 95%CI=0.09–0.34, p=0.001), involvement in school/work (studies=6, n=1,743, RR=1.13, 95%CI=1.03–1.24, p=0.012; NNT=17.8), and quality of life (studies=4, n=505, SMD=0.23, 95%CI=0.004–0.456, p=0.046). Superiority of CSC regarding all outcomes was also evident at 6, 9–12, and 18–24 months of treatment (except general symptoms and depression at 18–24 months).

**Discussion:**

In early psychosis, CSC is superior to TAU across all meta-analyzable, highly relevant outcomes with small-to-medium effect sizes. These results support the need for funding and utilization of CSC in patients with early-phase psychosis.

